# Comparison of three digestive tract reconstruction methods for the treatment of Siewert II and III adenocarcinoma of esophagogastric junction: a prospective, randomized controlled study

**DOI:** 10.1186/s12957-019-1762-x

**Published:** 2019-12-06

**Authors:** Zhiguo Li, Jianhong Dong, Qingxing Huang, Wanhong Zhang, Kai Tao

**Affiliations:** Department of Minimal Invasive Digestive Surgery, Shanxi Tumor Hospital, Shanxi Medical University, Taiyuan, 030013 China

**Keywords:** Piggyback jejunal interposition reconstruction single-tract reconstruction, Piggyback jejunal interposition reconstruction double-tract reconstruction, Total gastrectomy esophageal jejunal Roux-en-Y anastomosis, Reflux esophagitis

## Abstract

**Background:**

The incidence of adenocarcinoma of esophagogastric junction (AEG) has recently risen worldwide, including in Eastern Asia. The aim of the study was to explore the short-term and long-term clinical efficacy of piggyback jejunal interposition reconstruction single-tract reconstruction (PJIRSTR), piggyback jejunal interposition reconstruction double-tract reconstruction (PJIRDTR), and total gastrectomy esophageal jejunal Roux-en-Y anastomosis (TGRY) for the treatment of Siewert II and III AEG patients.

**Methods:**

A total of 300 Siewert II and III AEG patients admitted to Shanxi Tumor Hospital from June 2015 to December 2017 were prospectively selected. Patients were randomly divided into PJIRSTR group (*n* = 98), PJIRDTR group (*n* = 103), and TGRY group (*n* = 99) using the random number table method.

**Results:**

There were no statistically significant differences in total operation time, intraoperative blood loss, time of first anal exhaust, and postoperative hospital stay among the three groups (*F* = 2.526, 0.457, 0.234, 0.453; *P >* 0.05). The reconstruction time of PJIRSTR group and PJIRDTR group was longer than that of TGRY group (*P* < 0.01). There were no significant differences in cases of anastomotic leakage, anastomotic bleeding, abdominal infection, incision infection, ileus, and dumping syndrome in three groups (*P* > 0.05). The incidence of reflux esophagitis at 3, 6, 12, and 18 months after surgery in the PJIRSTR group and the PJIRDTR group were significantly lower than TGRY group in the same period (*P* < 0.05). Compared with PJIRDTR group and TGRY group, PJIRSTR group had a small fluctuation range of postoperative nutrition indexes and had basically recovered to the preoperative level at 18 months. Four patients of Visick grade IV presented in TGRY group 18 months postoperatively, which was significantly higher compared with the other two groups.

**Conclusion:**

Compared with PJIRDTR and TGRY, PJIRSTR can significantly reduce the incidence of postoperative reflux esophagitis and improve the long-term nutritional status of patients.

**Trial registration:**

Chinese Clinical Trial Registry, ChiCTR-IIR-16007733. Registered 07 November 2015 – Retrospectively registered, http://www.chictr.org.cn/searchproj.aspx.

## Background

Adenocarcinoma of esophagogastric junction (AEG) refers to the adenocarcinoma that occurs in the esophagogastric junction and within the range of 5 cm in both directions. In recent years, the incidence of AEG in eastern and western countries is increasing year by year [1].

At present, there is still a great controversy about the methods of digestive tract reconstruction for the treatment of AEG. European and American scholars believe that total gastrectomy (TG) should be selected since proximal gastrectomy (PG) may lead to the recurrence of gastric cancer due to cardiac loss, high incidence of long-term complications, and incomplete lymph node dissection [2]. However, Japanese scholars believe that PG should be selected because retention of residual stomach not only stores part of food and water but also ensures the absorption of nutrients such as iron, thus reducing the incidence of anemia and other complications [3]. With more and more in-depth studies on AEG, the views of Italian scholars have been accepted by the vast majority of scholars, who believe that there is no significant correlation between the survival rate of AEG patients and whether they receive PG or TG [4].

Proximal gastrectomy combined with jejunal interposition reconstruction has become an ideal choice for the treatment of for Siewert II and III AEG, since studies have shown that it can ensure the surgical safety, achieve an ideal radical cure, and achieve comparable 5-year overall survival with TG [5–7]. In China, piggyback jejunal interposition reconstruction (PJIR), proposed by Shanxi Tumor Hospital, was performed on the basis of Roux-en-Y jejunal anastomosis on the jejunum of the esophagus, and then jejunal remnant jejunostomy was performed on the appropriate location of jejunum loop. PJIR is divided into single-tract reconstruction (STR) and double-tract reconstruction (DTR), and its efficacy has been preliminarily verified [8]. However, there is still no consensus on the method of digestive tract reconstruction for Siewert II and III AEG patients.

This study prospectively analyzed the clinical pathological data of 300 Siewert II and III AEG patients and discussed the clinical effect of piggyback jejunal interposition reconstruction single-tract reconstruction (PJIRSTR), piggyback jejunal interposition reconstruction double-tract reconstruction (PJIRDTR), and total gastrectomy esophageal jejunal Roux-en-Y anastomosis (TGRY) for the treatment for AEG.

## Methods

### Design

We conducted a single-center, prospective, interventional, randomized therapeutic clinical trial to discuss the clinical effect of PJIRSTR, PJIRDTR, and TGRY for the treatment for AEG. This study was approved by the ethics committee of Shanxi Tumor Hospital. All patients signed informed consent and all surgeries were performed by the same surgeon. The study was registered at Chinese Clinical Trial Registry (http://www.chictr.org.cn/index.aspx; no. ChiCTR-IIR-16007733).

### Participants

About 300 patients with AEG admitted to Shanxi Tumor Hospital from June 2015 to December 2017 were selected. Inclusion criteria: Patients (1) aged 18 to 75 years; (2) with cT_l-3_N_0_M_0_ [TNM staging (AJCC 8th edition [8]) was used as the standard Siewert II or III type AEG]. The location of the AEG was defined as lower margin of palisading small vessels on endoscopy according to the Japanese Classification of Esophageal Cancer (11th edition) [9]; (3) tumor from lower dentate line ≤ 4 cm, the tumor diameter < 4 cm; (4) with primary tumors without distant metastasis; (5) with no surgical contraindications; and (6) with no history of malignant tumor surgery. Exclusion criteria: Patients (1) with other serious diseases that would not tolerate anesthesia and surgery, (2) had locally advanced or invasive carcinoma and (3) aged > 75 years old. About 300 patients were divided into PJIRSTR group (*n* = 98), PJIRDTR group (*n* = 103), and TGRY group (*n* = 99) according to the random number table.

### Surgical operation

Within 2 weeks after enrolment, surgeons performed surgery via the abdominal transhiatal (TH) approach, according to the study protocol. The upper abdominal midline incision was used, and D1^+^ or D2 lymph node dissection was performed. During the operation, the proximal and distal incisional margins of the resected specimens with suspicious positive incisional margins were sent for rapid pathology, and then the digestive tract reconstruction was performed.

**Fig. 1:**
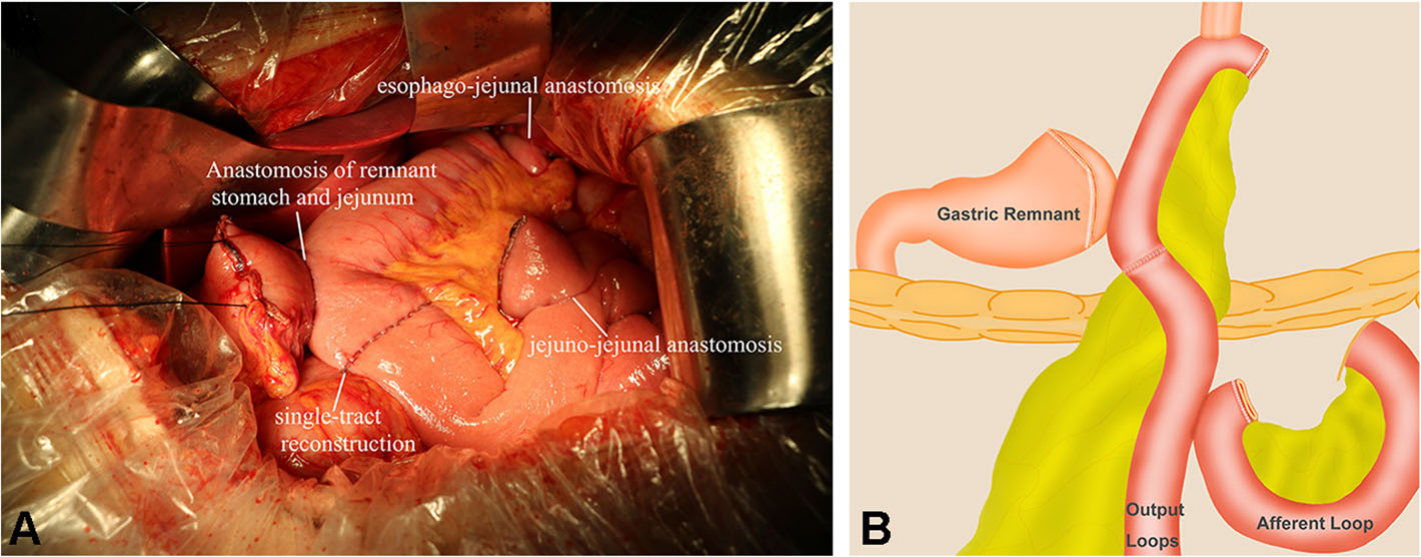
The piggyback jejunal interposition reconstruction single-tract reconstruction. **a** is the picture of the single-tract reconstruction of proximal gastrectomy with piggyback jejunal interposition reconstruction; (b) is the schematic diagram

**Fig. 2:**
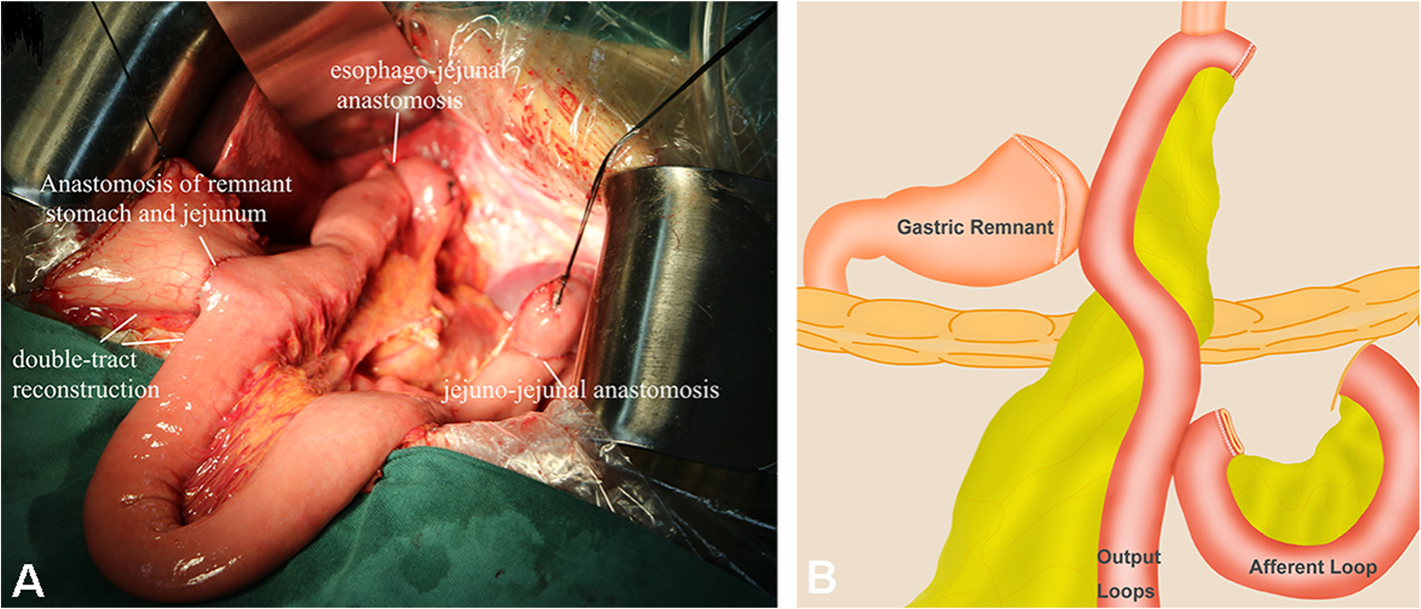
The piggyback jejunal interposition reconstruction double-tract reconstruction. **a** is the picture of the double-tract reconstruction of proximal gastrectomy with piggyback jejunal interposition reconstruction; (**b**) is the schematic diagram

For PJIRSTR group, jejunum was cut off about 20–25 cm from the Treitz ligament and was then lifted up to the end of the esophagus before or after the colon, followed by esophageal and jejunal anastomosis using 26# stapler. Lateral anastomosis was performed between the jejunum and the posterior wall of the remnant stomach about 12~15 cm from the esophageal jejunal anastomosis. The jejunum was closed with a closure device at 3 cm below the anastomosis to completely block the jejunum content passage. The jejunum was anastomosed with the proximal jejunum at the distal end of the ligation about 5~10 cm, as shown in Fig. 1. For PJIRDTR group, jejunum was not closed, but the distance between the gastrojejunal anastomosis and jejuno-jejunal anastomosis should be more than 30 cm to prevent reflux. The remaining surgical methods were the same as PJIRSTR group (Fig. 2). For TGRY group, the duodenal stump was closed, jejunum was cut off 20 cm from the distal end of the Treitz ligament, and the distal jejunum was anastomosed with the lower end of the esophagus. Meanwhile, proximal jejunum and distal jejunum were anastomosed about 40–45 cm from the distal end of the jejunal esophageal anastomosis. Three groups of patients were indwelling nutrition tube, not indwelling gastric tube. From the first day after operation, enteral nutrition support was performed with nutrient tube for 1 week, and 400 ml Nestle Healthcare Nutrition NUTREN OPTIMUM was infused daily; after 1 week, patients began to take oral nutrition supplement (Nestle Healthcare Nutrition NUTREN OPTIMUM, 1500 ml, daily).

### Observation indexes and Evaluation criteria

Operation time, digestive tract reconstruction time, intraoperative blood loss, first anal exhaust time, and postoperative hospital stay were recorded. Postoperative complications including anastomotic leakage, anastomotic bleeding, abdominal infection, incision infection, ileus, and dumping syndrome were observed. The postoperative pathological examinations showed that patients with positive lymph nodes or tumor invading all layers of gastric wall underwent the six-cycle SOX chemotherapy.

### Postoperative nutritional status and endoscopic gastroesophageal reflux

All the patients in three groups were followed up to 30 months after surgery, and the patients were followed up by telephone and returned to the hospital regularly for review. Visick score [8] was used to evaluate gastroesophageal reflux symptoms 18 months postoperatively: Visick grade I, asymptomatic; Visick grade II, occasional symptoms; Visick grade III, apparent but tolerable symptoms; and Visick grade IV, apparent and intolerable symptoms.

Nutritionist, specialist nurses, and physicians were involved, and nutritional adjustment and postoperative guidance were performed during follow-up of patients. Patients included were given medical cost reduction during follow-up; therefore the compliance is good. Body weight and the nutritional status including hemoglobin, total serum protein, serum albumin, and blood vitamin B12 were accessed at preoperative and postoperative 3, 6, 12, and 18 months. Esophagitis was assessed using fiber-optic endoscopy, and the incidence of reflux esophagitis was observed at postoperative 3, 6, 12, and 18 months. All patients in the three groups underwent postoperative endoscopy at 3, 6, 12, and 18 months to evaluate gastroesophageal reflux.

### Statistical methods

Statistical analysis was performed using SPSS 19.0 software, and values are presented as mean ± standard deviation (SD) or numbers (percentage). Between-group comparisons were analyzed by one-way ANOVA for normally distributed data. Categorical data were analyzed with the Chi-square test. *P* < 0.05 was considered statistically significant.

## Results

### Patient characteristics

Baseline characteristics of treatment groups were well balanced (Table 1) (*P* > 0.05).

**Table 1:** General information

Variables (*n*, %)	PJIRSTR (*n* = 98)	PJIRDTR (*n* = 103)	TGRY (*n* = 99)	*P* value
Male	88 (89.80)	90 (87.38)	82 (82.83)	0.345
Age (years)				0.458
< 60	25 (25.51)	23 (22.33)	18 (18.18)	
≥ 60	73 (74.49)	80 (77.67)	18 (18.18)	
Differentiation				0.572
High-middle	60 (61.22)	70 (67.96)	66 (66.67)	
Low	38 (38.78)	33 (32.04)	33 (33.33)	
TNM stage				0.562
I	81 (82.65)	87 (84.47)	78 (78.79)	
II	17 (17.35)	16 (15.53)	21 (21.21)	
Siewert type				0.628
II	45 (45.92)	48 (46.60)	40 (40.40)	
III	53 (54.08)	55 (53.40)	59 (59.60)	
Clinical stage				0.562
T1N0M0 stage IA	61 (62.24)	65 (63.11)	63 (63.64)	
T2N0M0 stage IB	20 (20.41)	22 (21.36)	15 (15.15)	
T3N0M0 stage II	17 (17.35)	16 (15.53)	21 (21.21)	

### Surgical outcomes

No significant differences were observed in operation time, intraoperative blood loss, first anal exhaust time, and postoperative hospital stay among the three groups (*P* > 0.05). No patient needed combined organ resection among the three groups. There were significantly fewer lymph nodes in the PJIRSTR and PJIRDTR than in the TGRY, whereas R0 resection was performed in all patients. As shown in Table 2, the reconstruction time of PJIRSTR group and PJIRDTR group was longer than that of TGRY group (*P* < 0.01) since piggyback jejunal interposition reconstruction (PJIRSTR group and PJIRDTR group) involves the trimming of the residual stomach and anastomosis of the residual stomach and jejunum. No significant differences were noted in the early complication rates regarding anastomotic leakage, anastomotic bleeding, abdominal infection, incision infection, ileus, and dumping syndrome among three groups (*P* > 0.05) (Table 3). Among them, two patients with anastomotic leakage were fully drained through the abdominal drainage tube and recovered after enteral and parenteral nutrition support. One patient with abdominal infection and two patients with incision infection complicated with incision fat liquefaction were treated by subcutaneous drainage and recovered. One case of intestinal obstruction after total gastrectomy was treated with internal conservative therapy, which was ineffective. Thus a second operation was performed and recovered. Of 300 patients, 10 underwent 6-cycle SOX chemotherapy, including 7 with perigastric lymph node metastasis and 3 with tumor invading all layers of gastric wall. In the PJIRSTR group, none of the patients developed cancer recurrence, but one patient died of acute pulmonary infarction. In the PJIRDTR group, one patient died of pneumonia without gastric cancer recurrence. In the TGRY group, one patient with pathological T4aN1M0 stage IIIA developed retroperitoneal lymph node metastasis but is currently alive.

**Table 2:** Comparison of intraoperative and postoperative conditions among three groups

	PJIRSTR (*n* = 98)	PJIRDTR (*n* = 103)	TGRY (*n* = 99)	*P* value
Lymph node dissection (*n*, %)				0.017
D1 +	98	103	95	
D2	0	0	4	
Combined resection (*n*, %)				-
Gall bladder	0	0	0	
Spleen	0	0	0	
Lymph node dissection number	22 ± 3	23 ± 3	37 ± 4	< 0.001
R0 resection (*n*, %)	98 (100)	103 (100)	99 (100)	-
Operation time (min)	144.37 ± 2.51	143.62 ± 2.98	143.29 ± 4.51	0.082
Digestive tract reconstruction time (min)	53.85 ± 4.51	50.22 ± 3.82	31.14 ± 5.23	<0.01
Intraoperative blood loss (ml)	139.09 ± 8.85	140.22 ± 7.93	139.78 ± 8.52	0.634
First anal exhaust time (h)	59.95 ± 5.09	60.04 ± 6.53	60.49 ± 6.31	0.791
Postoperative hospital stay (d)	10.53 ± 1.31	10.52 ± 1.18	10.68 ± 1.35	0.636

**Table 3:** Comparison of postoperative complications among three groups

Complications (*n*, %)	PJIRSTR (*n* = 98)	PJIRDTR (*n* = 103)	TGRY (*n* = 99)	*P* value
				0.999
Anastomotic leakage	0	1 (0.97)	1 (1.01)	
Anastomotic bleeding	0	0	0	
Abdominal infection	1 (1.02)	0	0	
Incision infection	1 (1.02)	1 (0.97)	0	
Ileus	0	0	1 (1.01)	
Dumping syndrome	0	0	0	

### Postoperative nutritional status and reflux esophagitis

The incidence of reflux esophagitis at 3, 6, 12, and 18 months after surgery in the PJIRSTR group and the PJIRDTR group was significantly lower than TGRY group in the same period (*P* < 0.05) (Table 4). Patients with reflux esophagitis in PJIRSTR group and PJIRDTR group disappeared after oral administration of esomeprazole enteric-coated tablets (AstraZeneca) and encouragement of eating solid food and patients with alkaline reflux esophagitis in TGRY group were relieved after eating solid food and avoiding lying down.

**Table 4:** Comparison of the incidence of endoscopic gastroesophageal reflux among three groups

Time (*n*, %)	PJIRSTR (*n* = 98)	PJIRDTR (*n* = 103)	TGRY (*n* = 99)	*P* value
3 months	2 (2)	2 (1.9)	15 (15.2)	< 0.01
6 months	2 (2)	3 (2.9)	18 (18.2)	< 0.01
12 months	3 (3.1)	3 (2.9)	18 (18.2)	< 0.01
18 months	3 (3.1)	3 (2.9)	20 (18.2)	< 0.01

**Fig. 3:**
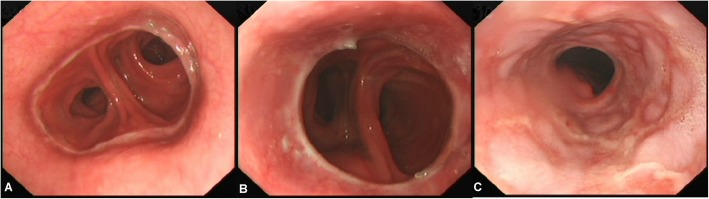
Gastroscopic examination of anastomotic site after (**a**) PJIRSTR, (**b**) PJIRDTR, and (**c**) TGRY

As shown in Table 5, there were no significant differences in patients of Visick grade I, Visick grade II, and Visick grade III in three groups (*P* > 0.05). There were four patients of grade IV Visick in TGRY group. After oral administration of mosapride citrate tablets (Jiangsu howson), two patients had remission of symptoms, while two patients had no remission thus had to be injected nutrition for a long time. The results of gastroscopy at postoperative 18 months were shown in Fig. 3.

**Table 5:** Gastroesophageal reflux symptom evaluation (Visick score) for three groups at postoperative 18 months

Visick grade (*n*, %)	PJIRSTR (*n* = 98)	PJIRDTR (*n* = 103)	TGRY (*n* = 99)	*P* value
I	94 (95.9)	94 (91.3)	84 (84.8)	0.03
II	3 (3.1)	6 (5.9)	7 (7.1)	0.44
III	1 (1.0)	3 (2.9)	4 (4.0)	0.42
IV	0	0	4 (4.0)	0.02

As shown in Table 6, the differences in body weight, hemoglobin, total serum protein, serum albumin, and blood vitamin B12 between the three groups at 3, 6, 12, and 18 months after surgery were statistically significant (*P* < 0.05). PJIRSTR group had a small fluctuation range of postoperative nutrition indexes and had basically recovered to the preoperative level at 18 months. The nutritional indexes in TGRY group fluctuated a lot postoperatively, especially the levels of hemoglobin and blood vitamin B12, which decreased gradually due to gastrectomy.

**Table 6:** Comparison of nutrition indexes among three groups

	PJIRSTR (*n* = 98)	PJIRDTR (*n* = 103)	TGRY (*n* = 99)	*P* value
Weight				
Preoperative	66.33 ± 3.116	66.10 ± 3.618	66.36 ± 3.824	0.844
Postoperative 3 months	59.54 ± 4.393	58.47 ± 4.385	57.36 ± 5.683	0.008
Postoperative 6 months	62.57 ± 3.407	60.80 ± 3.851	56.59 ± 4.936	< 0.001
Postoperative 12 months	63.17 ± 3.162	62.27 ± 3.425	57.84 ± 4.117	< 0.001
Postoperative 18 months	64.85 ± 3.020	64.08 ± 2.569	58.25 ± 4.246	< 0.001
Hemoglobin				
Preoperative	126.64 ± 5.562	128.06 ± 4.671	127.00 ± 5.449	0.137
Postoperative 3 months	120.24 ± 4.393	119.29 ± 4.807	114.48 ± 4.767	< 0.001
Postoperative 6 months	118.27 ± 4.123	117.40 ± 4.138	111.11 ± 5.099	< 0.001
Postoperative 12 months	120.98 ± 3.287	120.59 ± 3.382	115.72 ± 3.201	< 0.001
Postoperative 18 months	121.08 ± 3.289	120.56 ± 2.404	118.83 ± 3.207	< 0.001
Total serum protein				
Preoperative	72.12 ± 3.967	72.60 ± 3.861	72.69 ± 3.762	0.543
Postoperative 3 months	71.04 ± 3.548	71.40 ± 2.684	69.39 ± 2.494	< 0.001
Postoperative 6 months	68.36 ± 3.253	68.46 ± 3.183	65.34 ± 2.177	< 0.001
Postoperative 12 months	71.73 ± 2.595	70.73 ± 3.075	68.74 ± 1.747	< 0.001
Postoperative 18 months	71.45 ± 1.916	71.54 ± 1.984	70.09 ± 1.660	< 0.001
Serum albumin				
Preoperative	46.07 ± 2.412	45.50 ± 2.807	46.21 ± 3.354	0.183
Postoperative 3 months	44.09 ± 1.777	42.48 ± 2.244	42.63 ± 2.068	< 0.001
Postoperative 6 months	44.38 ± 1.897	43.81 ± 1.966	41.00 ± 1.922	< 0.001
Postoperative 12 months	44.76 ± 1.878	44.60 ± 2.157	42.84 ± 2.315	< 0.001
Postoperative 18 months	46.27 ± 2.952	44.00 ± 2.240	43.13 ± 2.207	< 0.001
Vitamin 12				
Preoperative	177.16 ± 5.807	176.05 ± 3.889	176.04 ± 5.443	0.248
Postoperative 3 months	170.26 ± 3.862	168.08 ± 3.741	157.02 ± 4.679	< 0.001
Postoperative 6 months	164.98 ± 3.956	154.13 ± 6.981	139.19 ± 4.690	< 0.001
Postoperative 12 months	167.87 ± 2.987	144.77 ± 6.588	114.86 ± 9.765	< 0.001
Postoperative 18 months	164.56 ± 3.840	147.16 ± 6.624	104.36 ± 9.494	< 0.001

## Discussion

For the surgical treatment of stage I and II gastric cancer, currently functional preservation is preferred to reduce long-term postoperative complications and improve the quality of life [10–12]. For patients with gastric cancer, choosing the appropriate method of digestive tract reconstruction directly determines the postoperative quality of life and nutritional status [13–16]. TGRY has been adopted for digestive tract reconstruction for nearly 10 years for Siewert II and III AEG patients. However, the development of PG has been limited due to the high incidence of reflux esophagitis and the decline of postoperative quality of life. After continuous improvement and development of surgical procedures, PJIR has become an ideal choice for the treatment of AEG. In addition, other methods including esophagogastrostomy (EG) [6, 17], jejunal interposition (JI) [18, 19], jejunal pouch interposition (JPI) [20, 21], and double tract (DT) [22, 23] are also available, among which EG is the most widely used due to simple operation [24]. However, severe reflux esophagitis is often associated with postoperative EG. Therefore, according to the location and size of the tumor, most surgeons currently perform total gastrectomy [25, 26], and a few surgeons adopt other reconstruction methods, such as JI, JPI, or DT, to minimize the occurrence of reflux esophagitis and other postoperative complications. In this study, Siewert II or III AEG patients were selected as the research objects to analyze the advantages and disadvantages of three methods of digestive tract reconstruction.

The results of this study showed that there were no significant differences in intraoperative blood loss, anal first exhaust time, and postoperative hospital stay among three groups. Although there were no significant differences in intraoperative and postoperative complications among the three types of digestive tract reconstruction, for the evaluation of postoperative reflux esophagitis and nutritional indicators, we found that PJIR had significant advantages in improving the nutritional status of patients and reducing the incidence of reflux esophagitis compared with TGRY. We speculated that one of the reasons for the lower incidence of reflux esophagitis of PJIRSTR and PJIRDTR group is that the jejunum interposition has an anti-reflux effect, making reflux fluid unable to flow or only a small amount of reflux into the esophagus, thereby reducing the incidence of reflux esophagitis or reducing reflux symptoms. In addition, the PJIRSTR group blocked the channel of proximal jejunal digestive fluid flowing back into the esophagus, which greatly reduces the occurrence of alkaline reflux esophagitis. Therefore, patients in PJIRSTR group had fewer patients of Visick grade II and III than PJIRDTR group, PJIRDTR group had fewer patients of Visick grade II and III than TGRY group, and TGRY group had patients of Visick grade IV. Nozaki [27] and Namikawa [28] reported that the incidence of reflux esophagitis does not significantly differ between the proximal gastrectomy with jejunal interposition and TGRY. However, in our study, four Visick grade IV patients with severe reflux esophagitis appeared in the TGRY group. We speculated that PJIRDTR provides double output channels for food transit, and this split transit approach can effectively prevent and reduce the incidence of esophageal reflux [29].

The function-preserving operation retained the distal stomach, increased the single-meal food intake, reduced the number of meals, and improved the quality of life [27]. Postoperative nutritional status at postoperative 3, 6, 12, and 18 months including body weight, hemoglobin, total serum protein, serum albumin, and blood vitamin B12 in PJIRSTR group was stable and reached the preoperative level by 18 months. It may be related to the fact that for PJIRSTR group, food can enter into the residual stomach through the interposition jejunum and was fully digested with gastric acid and then entered into the duodenum to stimulate the secretion of various hormones, which is more consistent with the physiological pathway of food. A retrospective study of 1061 cases of total gastrectomy, proximal gastrectomy, or distal gastrectomy in Japan [30] showed that PG had a better prognosis than TG and duodenal pathway reconstruction had a better prognosis than non-duodenal pathway. It is recommended to minimize gastrectomy and retain duodenal food flow pathway without residual cancer in the residual stomach. The decline of each index in PJIRDTR group was larger than that PJIRSTR group whereas was smaller than that in TGRY group. It is possible that most of the food in PJIRDTR group is discharged into the intestinal tract without full digestion, which thereby affects the absorption of food, increases the feeling of abdominal fullness, and reduces the food intake, eventually affecting the patient’s nutritional status [31–33]. Furthermore, the remnant gastric antrum provides the capacity for food storage, which not only delays emptying time to ensure the efficient mixing of food with the digestive juices but also promotes gastrin (GAS) secretion for adequate chymus digestion, ultimately enhancing patients’ long-term quality of life [17, 29]. Patients in TGRY group, on the other hand, were in poorer nutritional status than the other two groups because the food enter directly into the intestinal tract and there was no digestive function in the stomach [34]. Jung et al. [35] thought that the change rate of body weight in laparoscopic proximal gastrectomy with double-tract reconstruction (LPG-DT) group was significantly lower than in laparoscopic total gastrectomy (LTG) group. The serum vitamin B12 level in the LPG-DT group was significantly higher than in the LTG group. Research by Kim and his colleagues [36] observed that LPG-DT was beneficial with regard to the absorption of iron and vitamin B12 compared to LTG. A recent study has shown that body weight and skeletal muscle index reduction rates were lower in the LPG-DT group than in the LTG group [37]. Although distal stomach was retained in PJIRDTR group and PJIRSTR group and gastric mucosa could continue to produce internal factors for hematopoiesis, postoperative follow-up data showed a downward trend or even mild anemia of hemoglobin and vitamin B12 in the two groups, which may be related to the decrease in the number of gastric wall cells and changes in the acid and alkali environment in the stomach. VB12 deficiency, which causes megaloblastic anemia and a spectrum of neuropsychiatric disorders, is one of the common long-term nutritional sequelae after gastrectomy [35, 38]. The acidic environment of the stomach facilitates the breakdown of vitamin B12 that is bound to food. Intrinsic factor, which is released by parietal cells in the stomach, binds to vitamin B12 in the duodenum. This vitamin B12–intrinsic factor complex subsequently aids in the absorption of vitamin B12 in the terminal ileum [35]. Detailedly, the alkaline digestive fluid from jejunum in PJIRDTR group flows back into the residual stomach, causing the increase of PH value in the residual stomach and the production obstacle of internal factor. Therefore, lack of internal factor, B12, or tetrahydrofolate can cause the development of the nucleus to lag behind the development of the cytoplasm and eventually resulting in megaloblastic anemia [24].

Loss of the normal physiological function in TGRY group, due to the excision of the distal residual stomach, can make patients more prone to malnutrition and lose weight postoperatively. For PJIRDTR group and PJIRSTR group, retention of the distal stomach maintains the normal gastrointestinal anatomy and part of the physiological functions, which is beneficial to the digestion and absorption of postoperative nutrition and can also improve the postoperative tolerance of chemotherapy. Takiguchi et al. [39] evaluated subjective symptoms using a well-designed validated questionnaire and a post-gastrectomy syndrome assessment scale (PGSAS-45). Their data showed that PG was significantly improved over TG in terms of preventing body weight loss, the necessity for additional meals, diarrhea, and dumping.

The limitation of this study is that the survival rate of the patients was not statistically analyzed in this study, which needs to be further studied.

In summary, these three methods of digestive tract reconstruction are safe. PJIR is suitable for AEG patients of Siewert II and III. PJIRSTR is preferred since it had good anti-reflux effect, improved the long-term nutritional status and living quality, and the operation was not complicated. Multi-center and long-term follow-up studies remain to be done to clarify this finding.

## Data Availability

The data used to support the findings of this study are available from the corresponding author upon request.

## Abbreviations

AEG: Adenocarcinoma of esophagogastric junction
DT: Double tract
EG: Esophagogastrostomy
JI: Jejunal interposition
JPI: Jejunal pouch interposition
PG: Proximal gastrectomy
PJIR: Piggyback jejunal interposition reconstruction
PJIRDTR: Piggyback jejunal interposition reconstruction double-tract reconstruction
PJIRSTR: Piggyback jejunal interposition reconstruction single-tract reconstruction
TGRY: Total gastrectomy esophageal jejunal Roux-en-Y anastomosis
